# (*E*)-1-(4-Chloro­phen­yl)-3-[4-(diethyl­amino)phen­yl]prop-2-en-1-one^1^

**DOI:** 10.1107/S1600536809054683

**Published:** 2010-01-09

**Authors:** Thawanrat Kobkeatthawin, Suchada Chantrapromma, Hoong-Kun Fun

**Affiliations:** a Crystal Materials Research Unit, Department of Chemistry, Faculty of Science, Prince of Songkla University, Hat-Yai, Songkhla 90112, Thailand; b X-ray Crystallography Unit, School of Physics, Universiti Sains Malaysia, 11800 USM, Penang, Malaysia

## Abstract

The asymmetric unit of the title chalcone derivative, C_19_H_20_ClNO, contains two independent mol­ecules, which differ in the conformations of the ethyl groups in the diethyl­amino substituents. In the crystal, weak inter­molecular C—H⋯O hydrogen bonds link mol­ecules into ribbons propogating in [010]. The crystal packing also exhibits weak C—H⋯π inter­actions.

## Related literature

For bond-length data, see: Allen *et al.* (1987 ▶). For hydrogen-bond motifs, see: Bernstein *et al.* (1995 ▶). For related structures, see: Chantrapromma *et al.* (2009 ▶); Fun *et al.* (2009 ▶); Suwunwong *et al.* (2009 ▶). For background to and applications of chalcones, see: Svetlichny *et al.* (2007 ▶); Xu *et al.* (2005 ▶). For the stability of the temperature controller used in the data collection, see: Cosier & Glazer, (1986 ▶).
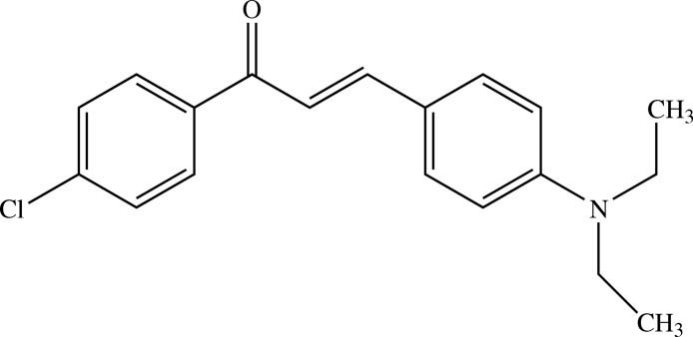


## Experimental

### 

#### Crystal data

C_19_H_20_ClNO
*M* *_r_* = 313.81
Monoclinic, 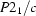

*a* = 17.2073 (2) Å
*b* = 11.9467 (1) Å
*c* = 15.7996 (2) Å
β = 97.268 (1)°
*V* = 3221.84 (6) Å^3^
*Z* = 8
Mo *K*α radiation
μ = 0.24 mm^−1^
*T* = 100 K
0.33 × 0.23 × 0.10 mm

#### Data collection

Bruker APEXII CCD area-detector diffractometer
Absorption correction: multi-scan (*SADABS*; Bruker, 2005 ▶) *T*_min_ = 0.925, *T*_max_ = 0.977
47596 measured reflections
9400 independent reflections
6241 reflections with *I* > 2σ(*I*)
*R*_int_ = 0.041

#### Refinement

*R*[*F* ^2^ > 2σ(*F* ^2^)] = 0.048
*wR*(*F* ^2^) = 0.122
*S* = 1.10
9400 reflections
401 parameters
H-atom parameters constrained
Δρ_max_ = 0.32 e Å^−3^
Δρ_min_ = −0.35 e Å^−3^

### 

Data collection: *APEX2* (Bruker, 2005 ▶); cell refinement: *SAINT* (Bruker, 2005 ▶); data reduction: *SAINT*; program(s) used to solve structure: *SHELXTL* (Sheldrick, 2008 ▶); program(s) used to refine structure: *SHELXTL*; molecular graphics: *SHELXTL*; software used to prepare material for publication: *SHELXTL* and *PLATON* (Spek, 2009 ▶).

## Supplementary Material

Crystal structure: contains datablocks global, I. DOI: 10.1107/S1600536809054683/cv2675sup1.cif

Structure factors: contains datablocks I. DOI: 10.1107/S1600536809054683/cv2675Isup2.hkl

Additional supplementary materials:  crystallographic information; 3D view; checkCIF report

## Figures and Tables

**Table 1:** Hydrogen-bond geometry (Å, °) *Cg*1 is the centroid of the C10*B*–C15*B* ring.

*D*—H⋯*A*	*D*—H	H⋯*A*	*D*⋯*A*	*D*—H⋯*A*
C1*B*—H1*BA*⋯O1*B*^i^	0.93	2.47	3.2746 (19)	145
C16*B*—H16*C*⋯O1*A*^ii^	0.97	2.42	3.3749 (19)	168
C2*A*—H2*AA*⋯*Cg*1^iii^	0.93	2.60	3.3377 (17)	137

Symmetry codes: (i) 
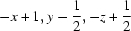
; (ii) 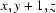; (iii) 
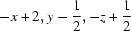
.

**Fig. 1.**
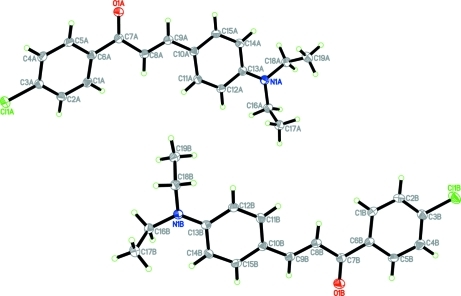
Two independent molecules of the title compound, with 50% probability displacement ellipsoids and the atom-numbering scheme.

**Fig. 2.**
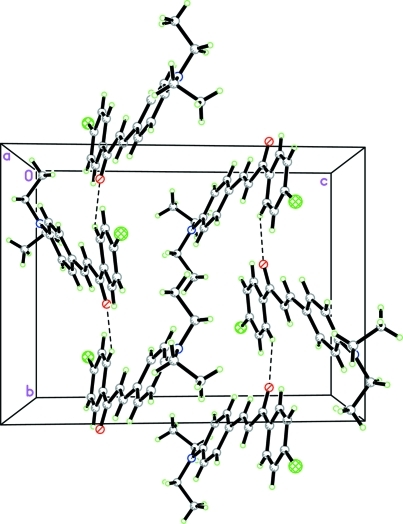
The crystal packing of the title compound view along the a-axis, showing ribbons running along the b axis. Hydrogen bonds are shown as dashed lines.

## supplementary crystallographic information

### Comment

In continuation of our study of chalcone derivatives (Chantrapromma *et al.*, 2009; Fun *et al.*, 2009; Suwunwong *et al*., 2009) which can be used for fluorescence probe for sensing of DNA or proteins (Svetlichny *et al.*, 2007; Xu *et al.*, 2005), the title compound (I) was synthesized and its fluorescence with the maximum emission at 437 nm was measured. We report here its crystal structure.

The asymmetric unit of (I) contains two molecules, *A* and *B*, respectively, which  differ in conformations of the ethyl groups of the diethylamino substituents. In molecule *A*, two ethyl groups are on the same side of the molecular plane, while they are on opposite sides in molecule *B* (Fig. 1). The bond lengths and bond angles in the two molecules are also slightly different. The molecules of (I) (Fig. 1) exist in an *E* configuration with respect to the C8═C9 double bond [1.349 (2) Å in molecule *A* and 1.341 (2) Å in molecule *B*] and the torsion angle C7–C8–C9–C10  is -178.39 (14)° in molecule *A* and 176.11 (4)° in molecule *B*. Two benzene rings are twisted at 16.27 (7)° in molecule *A* [16.99 (7) ° in molecule *B*]. The prop-2-en-1-one unit (C7–C9/O1) is planar with the rms 0.0066 (2) Å for molecule *A* [0.0116 (2) Å for molecule *B*]. The mean plane through the pro-2-en-1-one unit makes the dihedral angles of 19.02 (10) and 3.43 (10)° with the C1–C6 and C10–C15 benzene rings, respectively in molecule *A* [the corresponding values are 9.94 (10) and 7.31 (10)° in molecule *B*]. Two ethyl groups of the diethylamino substituent in molecule *A* are on the same side with the torsion angles C13A–N1A–C16A–C17A = -78.38 (18)° and C13A–N1A–C18A–C19A = 81.27 (18)° indicating the (-)-syn-clinal and (+)-syn-clinal conformations, respectively; whereas in molecule *B*, the two ethyl groups are on the opposite sides with the torsion angles C13B–N1B–C16B–C17B = 99.04 (17)° and C13B–N1B–C18B–C19B = 84.38 (17)° indicating the (+)-anti-clinal and (+)-syn-clinal conformations, respectively. Weak intramolecular C9A—H9AA···O1A, C5B—H5BA···O1B and C9B—H9BA···O1B hydrogen bonds generate S(5) ring motifs (Bernstein *et al.*, 1995). The bond distances in (I) are of normal values (Allen *et al.*, 1987) and are comparable with those in the related structure (Chantrapromma *et al.*, 2009).

In the crystal (Fig. 2), the 4-chlorophenyl and the pro-2-en-1-one units of the molecules are linked by weak intermolecular C—H···O hydrogen bonds (Table 1) resulting in the molecules being connected into ribbons propagating along the [0 1 0] direction. The crystal packing exhibits also weak C—H···π interactions (Table 1); Cg1 is the centroid of the C10B–C15B ring.

### Experimental

The title compound was synthesized by the condensation of 4-chloroacetophenone (0.40 g, 3 mmol) with 4-diethylaminobenzaldehyde (0.5 g, 3 mmol) in ethanol (20 ml) in the presence of 20% NaOH (aq) (5 ml). After stirring for 3 hr at 278 K, the resulting yellow solid was obtained and then collected by filtration, washed with distilled diethyl ether, dried and purified by repeated recrystallization from acetone. Yellow block-shaped single crystals of the title compound suitable for *x*-ray structure determination were recrystalized from methanol by the slow evaporation of the solvent at room temperature after several days, Mp. 374-375 K.

### Refinement

All H atoms were positioned geometrically and allowed to ride on their parent atoms, with d(C-H) = 0.93 Å for aromatic and CH, 0.97 Å for CH_2_ and 0.96 Å for CH_3_ atoms. The *U*_iso_ values were constrained to be 1.5*U*_eq_ of the carrier atom for methyl H atoms and 1.2*U*_eq_ for the remaining H atoms. A rotating group model was used for the methyl groups. The highest residual electron density peak is located at 0.68 Å from C6B and the deepest hole is located at 0.65 Å from Cl1B.

### Crystal data

**Table:**

C_19_H_20_ClNO	*F*(000) = 1328
*M**_r_* = 313.81	*D*_x_ = 1.294 Mg m^−^^3^
Monoclinic, *P*2_1_/*c*	Melting point = 374–375 K
Hall symbol: -P 2ybc	Mo *K*α radiation, λ = 0.71073 Å
*a* = 17.2073 (2) Å	Cell parameters from 9400 reflections
*b* = 11.9467 (1) Å	θ = 1.9–30.0°
*c* = 15.7996 (2) Å	µ = 0.24 mm^−^^1^
β = 97.268 (1)°	*T* = 100 K
*V* = 3221.84 (6) Å^3^	Block, yellow
*Z* = 8	0.33 × 0.23 × 0.10 mm

### Data collection

**Table:**

Bruker APEXII CCD area-detector diffractometer	9400 independent reflections
Radiation source: sealed tube	6241 reflections with *I* > 2σ(*I*)
graphite	*R*_int_ = 0.041
φ and ω scans	θ_max_ = 30.0°, θ_min_ = 1.2°
Absorption correction: multi-scan (*SADABS*; Bruker, 2005)	*h* = −23→24
*T*_min_ = 0.925, *T*_max_ = 0.977	*k* = −12→16
47596 measured reflections	*l* = −21→22

### Refinement

**Table:**

Refinement on *F*^2^	Primary atom site location: structure-invariant direct methods
Least-squares matrix: full	Secondary atom site location: difference Fourier map
*R*[*F*^2^ > 2σ(*F*^2^)] = 0.048	Hydrogen site location: inferred from neighbouring sites
*wR*(*F*^2^) = 0.122	H-atom parameters constrained
*S* = 1.10	*w* = 1/[σ^2^(*F*_o_^2^) + (0.0474*P*)^2^ + 0.7024*P*] where *P* = (*F*_o_^2^ + 2*F*_c_^2^)/3
9400 reflections	(Δ/σ)_max_ = 0.001
401 parameters	Δρ_max_ = 0.32 e Å^−^^3^
0 restraints	Δρ_min_ = −0.35 e Å^−^^3^

### Special details

**Table:**

Experimental. The crystal was placed in the cold stream of an Oxford Cryosystems Cobra open-flow nitrogen cryostat (Cosier & Glazer, 1986) operating at 100.0 (1) K.
Geometry. All esds (except the esd in the dihedral angle between two l.s. planes) are estimated using the full covariance matrix. The cell esds are taken into account individually in the estimation of esds in distances, angles and torsion angles; correlations between esds in cell parameters are only used when they are defined by crystal symmetry. An approximate (isotropic) treatment of cell esds is used for estimating esds involving l.s. planes.
Refinement. Refinement of F^2^ against ALL reflections. The weighted R-factor wR and goodness of fit S are based on F^2^, conventional R-factors R are based on F, with F set to zero for negative F^2^. The threshold expression of F^2^ > 2sigma(F^2^) is used only for calculating R-factors(gt) etc. and is not relevant to the choice of reflections for refinement. R-factors based on F^2^ are statistically about twice as large as those based on F, and R- factors based on ALL data will be even larger.

### Fractional atomic coordinates and isotropic or equivalent isotropic displacement parameters (Å^2^)

**Table:**

	*x*	*y*	*z*	*U*_iso_*/*U*_eq_	
Cl1A	1.33545 (2)	0.22869 (4)	0.37652 (3)	0.02632 (11)	
O1A	1.00783 (6)	−0.07143 (9)	0.29241 (7)	0.0250 (3)	
N1A	0.62274 (7)	0.22634 (11)	−0.01499 (8)	0.0220 (3)	
C1A	1.11964 (9)	0.17759 (13)	0.25803 (10)	0.0203 (3)	
H1AA	1.0859	0.2119	0.2151	0.024*	
C2A	1.19351 (9)	0.22284 (13)	0.28205 (10)	0.0209 (3)	
H2AA	1.2099	0.2857	0.2544	0.025*	
C3A	1.24235 (9)	0.17306 (13)	0.34767 (10)	0.0193 (3)	
C4A	1.21866 (9)	0.07958 (13)	0.38999 (10)	0.0216 (3)	
H4AA	1.2513	0.0484	0.4353	0.026*	
C5A	1.14600 (9)	0.03364 (13)	0.36369 (10)	0.0208 (3)	
H5AA	1.1305	−0.0304	0.3906	0.025*	
C6A	1.09523 (9)	0.08156 (13)	0.29733 (10)	0.0186 (3)	
C7A	1.01754 (9)	0.02632 (13)	0.27110 (10)	0.0185 (3)	
C8A	0.95504 (9)	0.09153 (13)	0.22182 (10)	0.0197 (3)	
H8AA	0.9640	0.1659	0.2086	0.024*	
C9A	0.88478 (9)	0.04481 (13)	0.19551 (9)	0.0193 (3)	
H9AA	0.8786	−0.0291	0.2118	0.023*	
C10A	0.81767 (9)	0.09526 (13)	0.14509 (10)	0.0190 (3)	
C11A	0.81717 (9)	0.20500 (13)	0.11330 (10)	0.0208 (3)	
H11A	0.8611	0.2498	0.1272	0.025*	
C12A	0.75375 (9)	0.24823 (13)	0.06228 (10)	0.0215 (4)	
H12A	0.7560	0.3214	0.0427	0.026*	
C13A	0.68503 (9)	0.18439 (13)	0.03865 (10)	0.0194 (3)	
C14A	0.68454 (9)	0.07499 (13)	0.07266 (10)	0.0220 (4)	
H14A	0.6400	0.0308	0.0606	0.026*	
C15A	0.74903 (9)	0.03261 (13)	0.12349 (10)	0.0207 (3)	
H15A	0.7469	−0.0401	0.1441	0.025*	
C16A	0.62152 (9)	0.34250 (13)	−0.04343 (10)	0.0236 (4)	
H16A	0.5841	0.3495	−0.0945	0.028*	
H16B	0.6728	0.3615	−0.0586	0.028*	
C17A	0.60016 (10)	0.42641 (14)	0.02253 (11)	0.0268 (4)	
H17A	0.6049	0.5011	0.0013	0.040*	
H17B	0.6349	0.4173	0.0746	0.040*	
H17C	0.5472	0.4137	0.0333	0.040*	
C18A	0.55139 (9)	0.16108 (14)	−0.03719 (10)	0.0231 (4)	
H18A	0.5658	0.0839	−0.0462	0.028*	
H18B	0.5244	0.1890	−0.0906	0.028*	
C19A	0.49514 (10)	0.16418 (15)	0.02990 (11)	0.0290 (4)	
H19A	0.4499	0.1194	0.0112	0.043*	
H19B	0.4792	0.2400	0.0380	0.043*	
H19C	0.5209	0.1351	0.0828	0.043*	
Cl1B	0.16956 (2)	0.81186 (4)	0.18284 (3)	0.03390 (13)	
O1B	0.51160 (6)	1.07982 (9)	0.24066 (8)	0.0283 (3)	
N1B	0.90996 (7)	0.73741 (11)	0.47926 (8)	0.0193 (3)	
C1B	0.40036 (9)	0.82319 (13)	0.25395 (10)	0.0209 (3)	
H1BA	0.4401	0.7729	0.2726	0.025*	
C2B	0.32374 (9)	0.78546 (14)	0.23590 (10)	0.0227 (4)	
H2BA	0.3121	0.7102	0.2425	0.027*	
C3B	0.26505 (9)	0.86039 (14)	0.20805 (10)	0.0221 (4)	
C4B	0.28072 (9)	0.97338 (14)	0.19930 (10)	0.0250 (4)	
H4BA	0.2405	1.0234	0.1816	0.030*	
C5B	0.35717 (9)	1.01030 (13)	0.21739 (10)	0.0217 (3)	
H5BA	0.3682	1.0859	0.2115	0.026*	
C6B	0.41803 (9)	0.93639 (13)	0.24428 (9)	0.0176 (3)	
C7B	0.49926 (9)	0.98249 (13)	0.26100 (10)	0.0189 (3)	
C8B	0.56250 (9)	0.91209 (13)	0.30276 (10)	0.0207 (3)	
H8BA	0.5512	0.8404	0.3206	0.025*	
C9B	0.63645 (9)	0.94976 (13)	0.31555 (9)	0.0187 (3)	
H9BA	0.6447	1.0201	0.2932	0.022*	
C10B	0.70517 (9)	0.89533 (13)	0.35960 (10)	0.0180 (3)	
C11B	0.70277 (9)	0.79079 (13)	0.39960 (10)	0.0200 (3)	
H11B	0.6547	0.7551	0.3992	0.024*	
C12B	0.76888 (9)	0.73904 (13)	0.43942 (10)	0.0196 (3)	
H12B	0.7645	0.6699	0.4655	0.023*	
C13B	0.84368 (9)	0.78947 (13)	0.44136 (9)	0.0173 (3)	
C14B	0.84647 (9)	0.89580 (13)	0.40271 (10)	0.0186 (3)	
H14B	0.8943	0.9322	0.4034	0.022*	
C15B	0.77899 (9)	0.94644 (13)	0.36396 (10)	0.0189 (3)	
H15B	0.7826	1.0170	0.3399	0.023*	
C16B	0.98874 (9)	0.77527 (13)	0.46801 (10)	0.0198 (3)	
H16C	0.9861	0.8185	0.4157	0.024*	
H16D	1.0215	0.7104	0.4619	0.024*	
C17B	1.02667 (9)	0.84630 (14)	0.54171 (11)	0.0251 (4)	
H17D	1.0795	0.8637	0.5329	0.038*	
H17E	1.0269	0.8056	0.5941	0.038*	
H17F	0.9975	0.9144	0.5446	0.038*	
C18B	0.90602 (9)	0.63347 (13)	0.52717 (10)	0.0209 (3)	
H18C	0.8606	0.6359	0.5578	0.025*	
H18D	0.9523	0.6276	0.5689	0.025*	
C19B	0.90064 (10)	0.53012 (14)	0.47043 (11)	0.0259 (4)	
H19D	0.9030	0.4641	0.5053	0.039*	
H19E	0.9435	0.5298	0.4370	0.039*	
H19F	0.8520	0.5312	0.4332	0.039*	

### Atomic displacement parameters (Å^2^)

**Table:**

	*U*^11^	*U*^22^	*U*^33^	*U*^12^	*U*^13^	*U*^23^
Cl1A	0.0194 (2)	0.0273 (2)	0.0314 (2)	−0.00409 (16)	−0.00011 (16)	0.00009 (18)
O1A	0.0213 (6)	0.0216 (6)	0.0316 (7)	−0.0014 (5)	0.0018 (5)	0.0073 (5)
N1A	0.0195 (7)	0.0215 (7)	0.0240 (7)	0.0001 (6)	−0.0012 (6)	0.0034 (6)
C1A	0.0200 (8)	0.0212 (9)	0.0196 (8)	0.0020 (7)	0.0013 (6)	0.0031 (7)
C2A	0.0223 (8)	0.0182 (8)	0.0228 (8)	−0.0009 (7)	0.0049 (7)	0.0011 (7)
C3A	0.0166 (7)	0.0190 (9)	0.0221 (8)	−0.0004 (6)	0.0016 (6)	−0.0023 (7)
C4A	0.0209 (8)	0.0204 (9)	0.0227 (8)	0.0043 (7)	−0.0001 (7)	0.0013 (7)
C5A	0.0219 (8)	0.0165 (8)	0.0241 (8)	0.0017 (6)	0.0039 (7)	0.0035 (7)
C6A	0.0198 (8)	0.0173 (8)	0.0192 (8)	0.0016 (6)	0.0040 (6)	0.0000 (6)
C7A	0.0189 (8)	0.0200 (9)	0.0173 (8)	0.0004 (6)	0.0048 (6)	0.0003 (6)
C8A	0.0205 (8)	0.0175 (8)	0.0214 (8)	0.0006 (6)	0.0035 (6)	0.0017 (6)
C9A	0.0218 (8)	0.0185 (8)	0.0185 (8)	0.0012 (6)	0.0053 (6)	0.0000 (6)
C10A	0.0193 (8)	0.0185 (9)	0.0193 (8)	0.0009 (6)	0.0033 (6)	−0.0008 (6)
C11A	0.0171 (8)	0.0203 (9)	0.0251 (8)	−0.0018 (6)	0.0034 (6)	−0.0019 (7)
C12A	0.0213 (8)	0.0166 (9)	0.0271 (9)	0.0009 (6)	0.0054 (7)	0.0030 (7)
C13A	0.0194 (8)	0.0199 (9)	0.0191 (8)	0.0008 (6)	0.0031 (6)	−0.0003 (6)
C14A	0.0205 (8)	0.0188 (9)	0.0258 (9)	−0.0027 (7)	0.0003 (7)	−0.0009 (7)
C15A	0.0229 (8)	0.0154 (8)	0.0231 (8)	−0.0007 (6)	0.0010 (7)	0.0007 (6)
C16A	0.0236 (8)	0.0234 (9)	0.0230 (8)	0.0002 (7)	−0.0007 (7)	0.0072 (7)
C17A	0.0237 (9)	0.0243 (10)	0.0328 (10)	−0.0003 (7)	0.0046 (7)	0.0045 (7)
C18A	0.0226 (8)	0.0231 (9)	0.0219 (8)	−0.0012 (7)	−0.0039 (7)	−0.0011 (7)
C19A	0.0265 (9)	0.0285 (10)	0.0317 (10)	−0.0065 (8)	0.0029 (8)	−0.0009 (8)
Cl1B	0.0241 (2)	0.0341 (3)	0.0408 (3)	−0.00895 (18)	−0.00643 (19)	0.0077 (2)
O1B	0.0260 (6)	0.0199 (7)	0.0384 (7)	−0.0013 (5)	0.0015 (5)	0.0073 (5)
N1B	0.0173 (7)	0.0173 (7)	0.0232 (7)	−0.0002 (5)	0.0016 (5)	0.0041 (5)
C1B	0.0232 (8)	0.0174 (9)	0.0222 (8)	0.0044 (7)	0.0032 (7)	0.0003 (7)
C2B	0.0281 (9)	0.0155 (8)	0.0243 (8)	−0.0030 (7)	0.0022 (7)	−0.0006 (7)
C3B	0.0204 (8)	0.0231 (9)	0.0222 (8)	−0.0033 (7)	0.0002 (7)	0.0008 (7)
C4B	0.0239 (9)	0.0219 (9)	0.0281 (9)	0.0042 (7)	−0.0014 (7)	0.0018 (7)
C5B	0.0251 (9)	0.0147 (8)	0.0247 (9)	−0.0005 (7)	0.0005 (7)	0.0000 (7)
C6B	0.0207 (8)	0.0170 (8)	0.0153 (7)	0.0013 (6)	0.0027 (6)	−0.0012 (6)
C7B	0.0213 (8)	0.0178 (9)	0.0178 (8)	0.0004 (6)	0.0029 (6)	0.0002 (6)
C8B	0.0226 (8)	0.0164 (8)	0.0229 (8)	0.0008 (6)	0.0025 (7)	0.0029 (7)
C9B	0.0217 (8)	0.0160 (8)	0.0191 (8)	0.0017 (6)	0.0049 (6)	−0.0015 (6)
C10B	0.0189 (8)	0.0163 (8)	0.0194 (8)	0.0014 (6)	0.0050 (6)	−0.0017 (6)
C11B	0.0170 (8)	0.0198 (9)	0.0242 (8)	−0.0012 (6)	0.0063 (6)	−0.0025 (7)
C12B	0.0215 (8)	0.0151 (8)	0.0225 (8)	−0.0005 (6)	0.0045 (6)	0.0006 (6)
C13B	0.0188 (8)	0.0166 (8)	0.0166 (7)	0.0004 (6)	0.0028 (6)	−0.0021 (6)
C14B	0.0176 (8)	0.0174 (8)	0.0209 (8)	−0.0032 (6)	0.0030 (6)	−0.0024 (6)
C15B	0.0229 (8)	0.0143 (8)	0.0202 (8)	0.0008 (6)	0.0054 (6)	−0.0011 (6)
C16B	0.0172 (8)	0.0197 (8)	0.0229 (8)	0.0010 (6)	0.0036 (6)	0.0013 (7)
C17B	0.0201 (8)	0.0257 (9)	0.0288 (9)	−0.0012 (7)	0.0002 (7)	−0.0004 (7)
C18B	0.0199 (8)	0.0207 (9)	0.0218 (8)	0.0003 (7)	0.0010 (6)	0.0041 (7)
C19B	0.0254 (9)	0.0198 (9)	0.0314 (9)	0.0011 (7)	−0.0007 (7)	0.0020 (7)

### Geometric parameters (Å, °)

**Table:**

Cl1A—C3A	1.7411 (15)	Cl1B—C3B	1.7405 (16)
O1A—C7A	1.2326 (18)	O1B—C7B	1.2320 (18)
N1A—C13A	1.3741 (19)	N1B—C13B	1.3687 (19)
N1A—C16A	1.458 (2)	N1B—C18B	1.460 (2)
N1A—C18A	1.459 (2)	N1B—C16B	1.4610 (19)
C1A—C2A	1.389 (2)	C1B—C2B	1.388 (2)
C1A—C6A	1.394 (2)	C1B—C6B	1.399 (2)
C1A—H1AA	0.9300	C1B—H1BA	0.9300
C2A—C3A	1.384 (2)	C2B—C3B	1.379 (2)
C2A—H2AA	0.9300	C2B—H2BA	0.9300
C3A—C4A	1.389 (2)	C3B—C4B	1.387 (2)
C4A—C5A	1.381 (2)	C4B—C5B	1.383 (2)
C4A—H4AA	0.9300	C4B—H4BA	0.9300
C5A—C6A	1.399 (2)	C5B—C6B	1.394 (2)
C5A—H5AA	0.9300	C5B—H5BA	0.9300
C6A—C7A	1.501 (2)	C6B—C7B	1.495 (2)
C7A—C8A	1.469 (2)	C7B—C8B	1.465 (2)
C8A—C9A	1.349 (2)	C8B—C9B	1.341 (2)
C8A—H8AA	0.9300	C8B—H8BA	0.9300
C9A—C10A	1.449 (2)	C9B—C10B	1.448 (2)
C9A—H9AA	0.9300	C9B—H9BA	0.9300
C10A—C15A	1.404 (2)	C10B—C11B	1.403 (2)
C10A—C11A	1.404 (2)	C10B—C15B	1.403 (2)
C11A—C12A	1.373 (2)	C11B—C12B	1.375 (2)
C11A—H11A	0.9300	C11B—H11B	0.9300
C12A—C13A	1.417 (2)	C12B—C13B	1.418 (2)
C12A—H12A	0.9300	C12B—H12B	0.9300
C13A—C14A	1.414 (2)	C13B—C14B	1.413 (2)
C14A—C15A	1.381 (2)	C14B—C15B	1.382 (2)
C14A—H14A	0.9300	C14B—H14B	0.9300
C15A—H15A	0.9300	C15B—H15B	0.9300
C16A—C17A	1.524 (2)	C16B—C17B	1.520 (2)
C16A—H16A	0.9700	C16B—H16C	0.9700
C16A—H16B	0.9700	C16B—H16D	0.9700
C17A—H17A	0.9600	C17B—H17D	0.9600
C17A—H17B	0.9600	C17B—H17E	0.9600
C17A—H17C	0.9600	C17B—H17F	0.9600
C18A—C19A	1.524 (2)	C18B—C19B	1.522 (2)
C18A—H18A	0.9700	C18B—H18C	0.9700
C18A—H18B	0.9700	C18B—H18D	0.9700
C19A—H19A	0.9600	C19B—H19D	0.9600
C19A—H19B	0.9600	C19B—H19E	0.9600
C19A—H19C	0.9600	C19B—H19F	0.9600
			
C13A—N1A—C16A	121.13 (13)	C13B—N1B—C18B	121.49 (12)
C13A—N1A—C18A	121.34 (13)	C13B—N1B—C16B	122.73 (13)
C16A—N1A—C18A	117.10 (12)	C18B—N1B—C16B	115.52 (12)
C2A—C1A—C6A	121.02 (14)	C2B—C1B—C6B	120.35 (14)
C2A—C1A—H1AA	119.5	C2B—C1B—H1BA	119.8
C6A—C1A—H1AA	119.5	C6B—C1B—H1BA	119.8
C3A—C2A—C1A	119.04 (15)	C3B—C2B—C1B	119.53 (15)
C3A—C2A—H2AA	120.5	C3B—C2B—H2BA	120.2
C1A—C2A—H2AA	120.5	C1B—C2B—H2BA	120.2
C2A—C3A—C4A	121.26 (14)	C2B—C3B—C4B	121.31 (15)
C2A—C3A—Cl1A	118.93 (12)	C2B—C3B—Cl1B	119.26 (13)
C4A—C3A—Cl1A	119.81 (12)	C4B—C3B—Cl1B	119.42 (12)
C5A—C4A—C3A	118.98 (15)	C5B—C4B—C3B	118.81 (15)
C5A—C4A—H4AA	120.5	C5B—C4B—H4BA	120.6
C3A—C4A—H4AA	120.5	C3B—C4B—H4BA	120.6
C4A—C5A—C6A	121.24 (15)	C4B—C5B—C6B	121.27 (15)
C4A—C5A—H5AA	119.4	C4B—C5B—H5BA	119.4
C6A—C5A—H5AA	119.4	C6B—C5B—H5BA	119.4
C1A—C6A—C5A	118.40 (14)	C5B—C6B—C1B	118.71 (14)
C1A—C6A—C7A	123.13 (14)	C5B—C6B—C7B	118.04 (14)
C5A—C6A—C7A	118.47 (14)	C1B—C6B—C7B	123.26 (14)
O1A—C7A—C8A	122.12 (14)	O1B—C7B—C8B	121.12 (14)
O1A—C7A—C6A	119.20 (14)	O1B—C7B—C6B	119.33 (14)
C8A—C7A—C6A	118.68 (14)	C8B—C7B—C6B	119.54 (14)
C9A—C8A—C7A	120.77 (15)	C9B—C8B—C7B	120.80 (15)
C9A—C8A—H8AA	119.6	C9B—C8B—H8BA	119.6
C7A—C8A—H8AA	119.6	C7B—C8B—H8BA	119.6
C8A—C9A—C10A	128.20 (15)	C8B—C9B—C10B	128.56 (15)
C8A—C9A—H9AA	115.9	C8B—C9B—H9BA	115.7
C10A—C9A—H9AA	115.9	C10B—C9B—H9BA	115.7
C15A—C10A—C11A	116.37 (14)	C11B—C10B—C15B	116.32 (14)
C15A—C10A—C9A	119.98 (14)	C11B—C10B—C9B	123.19 (14)
C11A—C10A—C9A	123.63 (14)	C15B—C10B—C9B	120.48 (14)
C12A—C11A—C10A	122.00 (14)	C12B—C11B—C10B	122.45 (14)
C12A—C11A—H11A	119.0	C12B—C11B—H11B	118.8
C10A—C11A—H11A	119.0	C10B—C11B—H11B	118.8
C11A—C12A—C13A	121.81 (15)	C11B—C12B—C13B	121.00 (14)
C11A—C12A—H12A	119.1	C11B—C12B—H12B	119.5
C13A—C12A—H12A	119.1	C13B—C12B—H12B	119.5
N1A—C13A—C14A	122.01 (14)	N1B—C13B—C14B	121.71 (14)
N1A—C13A—C12A	121.73 (14)	N1B—C13B—C12B	121.35 (14)
C14A—C13A—C12A	116.25 (14)	C14B—C13B—C12B	116.93 (14)
C15A—C14A—C13A	121.14 (14)	C15B—C14B—C13B	120.84 (14)
C15A—C14A—H14A	119.4	C15B—C14B—H14B	119.6
C13A—C14A—H14A	119.4	C13B—C14B—H14B	119.6
C14A—C15A—C10A	122.38 (15)	C14B—C15B—C10B	122.41 (14)
C14A—C15A—H15A	118.8	C14B—C15B—H15B	118.8
C10A—C15A—H15A	118.8	C10B—C15B—H15B	118.8
N1A—C16A—C17A	114.24 (13)	N1B—C16B—C17B	113.25 (13)
N1A—C16A—H16A	108.7	N1B—C16B—H16C	108.9
C17A—C16A—H16A	108.7	C17B—C16B—H16C	108.9
N1A—C16A—H16B	108.7	N1B—C16B—H16D	108.9
C17A—C16A—H16B	108.7	C17B—C16B—H16D	108.9
H16A—C16A—H16B	107.6	H16C—C16B—H16D	107.7
C16A—C17A—H17A	109.5	C16B—C17B—H17D	109.5
C16A—C17A—H17B	109.5	C16B—C17B—H17E	109.5
H17A—C17A—H17B	109.5	H17D—C17B—H17E	109.5
C16A—C17A—H17C	109.5	C16B—C17B—H17F	109.5
H17A—C17A—H17C	109.5	H17D—C17B—H17F	109.5
H17B—C17A—H17C	109.5	H17E—C17B—H17F	109.5
N1A—C18A—C19A	114.14 (13)	N1B—C18B—C19B	112.83 (13)
N1A—C18A—H18A	108.7	N1B—C18B—H18C	109.0
C19A—C18A—H18A	108.7	C19B—C18B—H18C	109.0
N1A—C18A—H18B	108.7	N1B—C18B—H18D	109.0
C19A—C18A—H18B	108.7	C19B—C18B—H18D	109.0
H18A—C18A—H18B	107.6	H18C—C18B—H18D	107.8
C18A—C19A—H19A	109.5	C18B—C19B—H19D	109.5
C18A—C19A—H19B	109.5	C18B—C19B—H19E	109.5
H19A—C19A—H19B	109.5	H19D—C19B—H19E	109.5
C18A—C19A—H19C	109.5	C18B—C19B—H19F	109.5
H19A—C19A—H19C	109.5	H19D—C19B—H19F	109.5
H19B—C19A—H19C	109.5	H19E—C19B—H19F	109.5
			
C6A—C1A—C2A—C3A	−1.8 (2)	C6B—C1B—C2B—C3B	0.2 (2)
C1A—C2A—C3A—C4A	−0.4 (2)	C1B—C2B—C3B—C4B	−1.3 (2)
C1A—C2A—C3A—Cl1A	179.03 (12)	C1B—C2B—C3B—Cl1B	178.31 (12)
C2A—C3A—C4A—C5A	2.3 (2)	C2B—C3B—C4B—C5B	1.3 (2)
Cl1A—C3A—C4A—C5A	−177.11 (12)	Cl1B—C3B—C4B—C5B	−178.31 (12)
C3A—C4A—C5A—C6A	−2.0 (2)	C3B—C4B—C5B—C6B	−0.2 (2)
C2A—C1A—C6A—C5A	2.1 (2)	C4B—C5B—C6B—C1B	−0.9 (2)
C2A—C1A—C6A—C7A	−177.12 (14)	C4B—C5B—C6B—C7B	178.88 (14)
C4A—C5A—C6A—C1A	−0.1 (2)	C2B—C1B—C6B—C5B	0.9 (2)
C4A—C5A—C6A—C7A	179.12 (14)	C2B—C1B—C6B—C7B	−178.86 (14)
C1A—C6A—C7A—O1A	161.56 (15)	C5B—C6B—C7B—O1B	−9.0 (2)
C5A—C6A—C7A—O1A	−17.6 (2)	C1B—C6B—C7B—O1B	170.70 (15)
C1A—C6A—C7A—C8A	−19.4 (2)	C5B—C6B—C7B—C8B	169.77 (14)
C5A—C6A—C7A—C8A	161.42 (14)	C1B—C6B—C7B—C8B	−10.5 (2)
O1A—C7A—C8A—C9A	−2.2 (2)	O1B—C7B—C8B—C9B	−3.8 (2)
C6A—C7A—C8A—C9A	178.77 (14)	C6B—C7B—C8B—C9B	177.38 (14)
C7A—C8A—C9A—C10A	−178.39 (14)	C7B—C8B—C9B—C10B	176.11 (14)
C8A—C9A—C10A—C15A	179.71 (15)	C8B—C9B—C10B—C11B	−2.2 (3)
C8A—C9A—C10A—C11A	1.6 (3)	C8B—C9B—C10B—C15B	177.64 (15)
C15A—C10A—C11A—C12A	−1.4 (2)	C15B—C10B—C11B—C12B	−1.5 (2)
C9A—C10A—C11A—C12A	176.80 (15)	C9B—C10B—C11B—C12B	178.41 (14)
C10A—C11A—C12A—C13A	−0.1 (2)	C10B—C11B—C12B—C13B	−0.4 (2)
C16A—N1A—C13A—C14A	174.59 (14)	C18B—N1B—C13B—C14B	173.21 (14)
C18A—N1A—C13A—C14A	2.3 (2)	C16B—N1B—C13B—C14B	−12.8 (2)
C16A—N1A—C13A—C12A	−6.0 (2)	C18B—N1B—C13B—C12B	−6.6 (2)
C18A—N1A—C13A—C12A	−178.37 (14)	C16B—N1B—C13B—C12B	167.43 (14)
C11A—C12A—C13A—N1A	−177.54 (14)	C11B—C12B—C13B—N1B	−178.57 (14)
C11A—C12A—C13A—C14A	1.9 (2)	C11B—C12B—C13B—C14B	1.6 (2)
N1A—C13A—C14A—C15A	177.16 (15)	N1B—C13B—C14B—C15B	179.29 (14)
C12A—C13A—C14A—C15A	−2.2 (2)	C12B—C13B—C14B—C15B	−0.9 (2)
C13A—C14A—C15A—C10A	0.8 (2)	C13B—C14B—C15B—C10B	−1.0 (2)
C11A—C10A—C15A—C14A	1.0 (2)	C11B—C10B—C15B—C14B	2.2 (2)
C9A—C10A—C15A—C14A	−177.26 (14)	C9B—C10B—C15B—C14B	−177.68 (14)
C13A—N1A—C16A—C17A	−78.38 (18)	C13B—N1B—C16B—C17B	99.04 (17)
C18A—N1A—C16A—C17A	94.27 (16)	C18B—N1B—C16B—C17B	−86.63 (16)
C13A—N1A—C18A—C19A	81.27 (18)	C13B—N1B—C18B—C19B	84.38 (17)
C16A—N1A—C18A—C19A	−91.36 (17)	C16B—N1B—C18B—C19B	−90.03 (16)

### Hydrogen-bond geometry (Å, °)

**Table:**

Cg1 is the centroid of the C10B–C15B ring.

**Table:**

*D*—H···*A*	*D*—H	H···*A*	*D*···*A*	*D*—H···*A*
C1B—H1BA···O1B^i^	0.93	2.47	3.2746 (19)	145
C16B—H16C···O1A^ii^	0.97	2.42	3.3749 (19)	168
C2A—H2AA···Cg1^iii^	0.93	2.60	3.3377 (17)	137

Symmetry codes: (i) −*x*+1, *y*−1/2, −*z*+1/2; (ii) *x*, *y*+1, *z*; (iii) −*x*+2, *y*−1/2, −*z*+1/2.

## References

[bb1] Allen, F. H., Kennard, O., Watson, D. G., Brammer, L., Orpen, A. G. & Taylor, R. (1987). *J. Chem. Soc. Perkin Trans. 2*, pp. S1–19.

[bb2] Bernstein, J., Davis, R. E., Shimoni, L. & Chang, N.-L. (1995). *Angew. Chem. Int. Ed. Engl* **34**, 1555–1573.

[bb3] Bruker (2005). *APEX2*, *SAINT* and *SADABS* Bruker AXS Inc., Madison, Wisconsin, USA.

[bb4] Chantrapromma, S., Suwunwong, T., Karalai, C. & Fun, H.-K. (2009). *Acta Cryst.* E**65**, o893–o894. 10.1107/S1600536809010496 PMC2968789 21582601

[bb5] Cosier, J. & Glazer, A. M. (1986). *J. Appl. Cryst.* **19**, 105–107.

[bb6] Fun, H.-K., Suwunwong, T., Boonnak, N. & Chantrapromma, S. (2009). *Acta Cryst.* E**65**, o2168–o2169. 10.1107/S1600536809031900 PMC2969873 21577575

[bb7] Sheldrick, G. M. (2008). *Acta Cryst.* A**64**, 112–122. 10.1107/S0108767307043930 18156677

[bb8] Spek, A. L. (2009). *Acta Cryst* D**65**, 148–155. 10.1107/S090744490804362X PMC2631630 19171970

[bb9] Suwunwong, T., Chantrapromma, S., Pakdeevanich, P. & Fun, H.-K. (2009). *Acta Cryst.* E**65**, o1575–o1576. 10.1107/S1600536809021850 PMC2969210 21582853

[bb10] Svetlichny, V. Y., Merola, F., Dobretsov, G. E., Gularyan, S. K. & Syrejshchikova, T. I. (2007). *J. Chem. Phys. Lipids*, **145**, 13–26. 10.1016/j.chemphyslip.2006.10.001 17125758

[bb11] Xu, Z., Bai, G. & Dong, C. (2005). *J. Bioorg. Med. Chem.* **13**, 5694–5699. 10.1016/j.bmc.2005.06.023 16006133

